# Sediment Microbial Communities and Their Potential Role as Environmental Pollution Indicators in Xuande Atoll, South China Sea

**DOI:** 10.3389/fmicb.2020.01011

**Published:** 2020-05-25

**Authors:** Biao Zhang, Yan Li, Shi-Zheng Xiang, Yu Yan, Rui Yang, Meng-Ping Lin, Xue-Mu Wang, Yu-Long Xue, Xiang-Yu Guan

**Affiliations:** ^1^School of Ocean Sciences, China University of Geosciences, Beijing, China; ^2^Marine Geological Survey Institute of Hainan Province, Haikou, China; ^3^Hebei Marine Resource Survey Center, Qinhuangdao, China

**Keywords:** environmental pollution indicator, microbial community, network, sediment, South China Sea

## Abstract

In this study, 39 sediment samples were collected from Qilian Island, Iltis Bank, and Yongxing Island in Xuande Atoll in the South China Sea (SCS), and the microbial community structures and distribution were analyzed. The microbial community was influenced by both natural environmental factors and human activities. The abundance of genera *Vibrio* and *Pseudoalteromonas*, which are associated with pathogenicity and pollutant degradation, were significantly higher in Qilian Island than in Yongxing Island and Iltis Bank, suggesting possible contamination of Qilian Island area through human activities. Pathogenic or typical pollutants-degrading bacteria were found to be negatively correlated with most of the commonly occurring bacterial populations in marine sediment, and these bacteria were more likely to appear in the sediment of deep water layer. This co-occurrence pattern may be due to bacterial adaptation to environmental changes such as depth and contaminations from human activities, including garbage disposal, farming, and oil spills from ships. The findings of this study could help in understanding the potential influences of human activities on the ecosystem at the microbial level.

## Introduction

Marine sediments are an important component of the marine environment and represent the largest organic carbon reservoir on Earth (Parkes et al., 2000). Marine sediments, which are mainly derived from continental transport and sedimentation of biological products, are rich in nutrients and provide important habitats for microorganisms (Kallmeyer et al., 2012; Parkes et al., 2014). Microorganisms in sediments play key roles in the regulation of major geochemical and eco-environmental processes of marine ecosystems, particularly nutrient dynamics and biogeochemical cycles (Fuhrman, 2009; Nogales et al., 2011; Graham et al., 2012). Environmental factors, such as pH, water temperature, silicate, and ocean currents, cause differences in the structure and diversity of marine microbial communities (Gilbert et al., 2009; Hollister et al., 2010; Kirchman et al., 2010). Besides, the dispersion and movement of microorganisms are also driven by hydrography (Hamdan et al., 2013). The grain-size distribution of sediments can reflect the hydrodynamic conditions, such as tide and ocean current. Bimodal distribution and large difference of sediment particle size indicate complex hydrodynamic forces (Anthony and Héquette, 2007; Hu et al., 2012).

The microbial community composition of sediment samples has been noted to show geographic differences. Examination of the microbial community from several marginal waters and sediments of the Sea of Japan, South China Sea (SCS) Trough, Sea of Okhotsk, Peruvian Marginal Sea, revealed that the structure and diversity of microbial communities vary among different regions (Rochelle et al., 1994; Inagaki et al., 2003; Newberry et al., 2004; Webster et al., 2006). For instance, the proportions of some genera belonging to the phyla *Proteobacteria*, *Firmicutes*, *Actinobacteria*, and *Bacteroides*, which can use petroleum as carbon sources, are increased in coastal sediments that are heavily polluted by human activities such as Hangzhou Bay and Bohai Bay; in addition, classes such as *γ-Proteobacteria* and *δ-Proteobacteria* have been found to dominate such areas (Kochling et al., 2011; Wang et al., 2013; Guan et al., 2014; Lu et al., 2016; Su et al., 2018). These microorganisms are considered to be potential indicator groups (Xiong et al., 2014). In addition, genera such as *Firmicutes* and *Bacilli* have also been noted to be dominant in many areas that have been heavily contaminated and disturbed by human activities (Zhang et al., 2008; Lu et al., 2016). Microbial communities in marine sediments are significantly influenced by changes in spatial distribution and environment factors, and specific microorganisms can be used as environmental indicators (Gillan et al., 2005; Glasl et al., 2019).

The SCS is located in the south of mainland China, and is exposed to complex tidal and ocean currents (Lien et al., 2005; Liu et al., 2010; Xiaodong Deng and Cai, 2013), which might affect migration and the distribution of microbial communities. Microbial communities in the sediments of the SCS are affected by both natural and anthropogenic factors. In the SCS, the sediment microbial diversity is high, and *γ-Proteobacteria*, *Planctomycetes* and *Bacteroidetes* are the main taxa in this area. Moreover, new unnamed bacteria are constantly being identified and different sediments have been shown to have different microbial community structures (Zhu et al., 2013; Yu et al., 2018). There are a large number of protease-producing bacteria in *γ-Proteobacteria*, which play an important role in the degradation of organic nitrogen in sediments (Zhou et al., 2009). The composition and diversity of microbial communities have been noted to show spatial and seasonal changes in the SCS (Du et al., 2011), and total organic carbon and nitrogen also impact bacterial community structure (Li and Wang, 2014). The hypoxic environment of the seabed forms a unique microbial community in which the anaerobic group of the community is more active (Walsh et al., 2016). In recent years, human activities and offshore oil spills have led to serious threats to the marine ecological environment, as well as causing significant changes to the structure and diversity of microbial communities in this region (Yuan, 2015; Limei Guo et al., 2017; Lufei Yang and Li, 2019).

Xuande Atoll is the largest residential area in the SCS, and is possibly influenced by human activities such as garbage disposal, farming, and oil spills from ships. In the present study, different sediment samples from three island reefs in Xuande Atoll area were investigated to elucidate the structure of the sediment microbial community, microbial response to human activities and environmental factors, and the role of the microbial community as indicators of pollution. The goals of this study were: (1) to identify the spatial distribution of microbial community structure in sediments among different islands and reefs, and the main impacts of various environmental factors on microbial communities; (2) to understand the role of sediment microorganisms in the SCS as indicators of environmental pollution associated with human activities; and (3) to reveal the possible response mechanism of sediment microbial communities to natural and anthropogenic factors. The results of this study will provide a reference for future environmental protection in the SCS, as well as impart valuable information on microbial indicators in the SCS.

## Materials and Methods

### Sample Collection and Grouping

**FIGURE 1:**
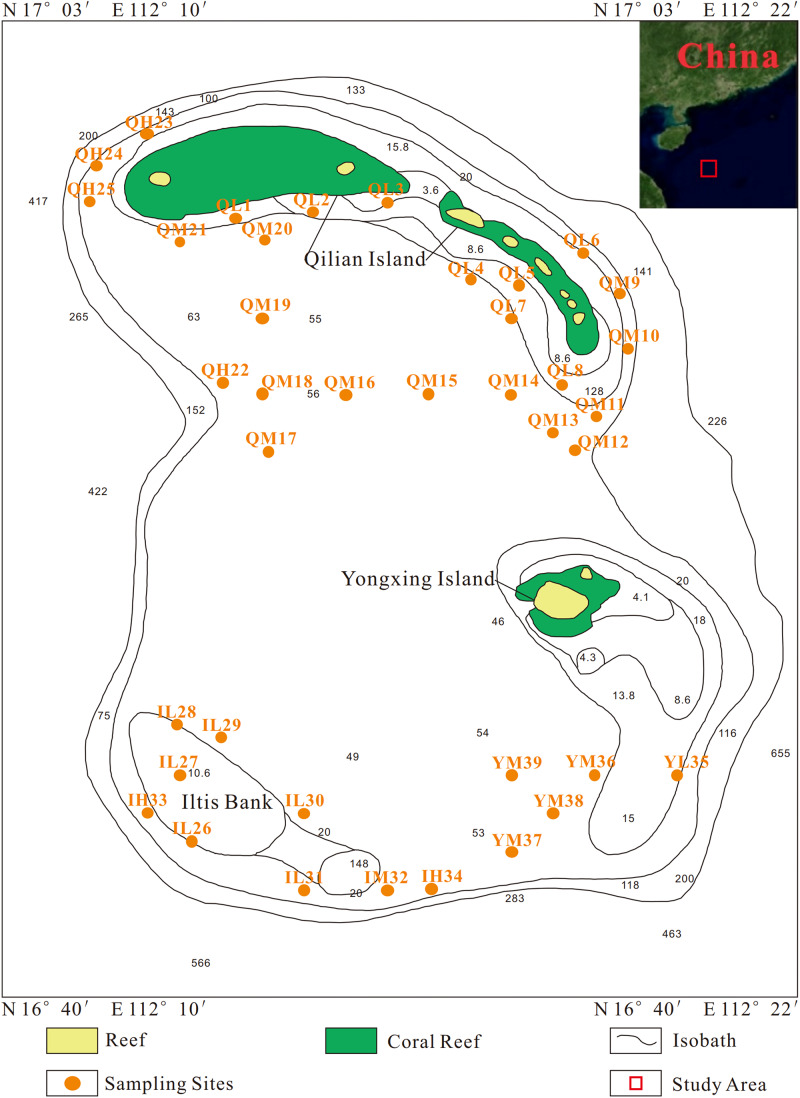
Sampling sites of all sediment samples.

A total of 39 surface sediment samples were collected from the Xuande Atoll area of the SCS using a grab sampler from September to October of 2015. The water depth of the sampling points ranged from 16.5 to 97.6 m. To study the microbial community structure among sediments at different water depths, the samples were divided into three groups, namely, L (15–50 m), M (50–70 m), and H (70–100 m) according to the water depth. The sediment samples with three depth layers were located at Qilian Island (QL1-8, QM9-21, QH22-25), Iltis Bank (IL26-31, IM32, IH33-34), and Yongxing Island (YL35, YM36-39) (Figure 1 and Table 1). To investigate the distribution of the regional microbial communities and the differences in microbial community structure among the three islands, all 39 samples were divided into three groups according to the distribution of sampling points on the three islands and three depth groups (Tables 2, 3). The collected sediment samples were quickly packed in 50 mL sterile centrifuge tubes and frozen at −20°C until they were transported to the laboratory, where they were stored at −80°C, and the genomic DNA was extracted as soon as possible.

**TABLE 1:** Sampling locations and depths.

Station	Longitude (E)	Latitude (N)	Depth (m)	Station	Longitude (E)	Latitude (N)	Depth (m)
QL1	112°14′09.208″	16°57′49.318″	43.0	QM21	112°13′04.165″	16°57′21.176″	60.0
QL2	112°15′37.499″	16°57′56.207″	34.7	QH22	112°13′48.754″	16°54′29.501″	73.6
QL3	112°17′03.659″	16°57′19.796″	18.1	QH23	112°12′26.245″	16°59′31.442″	77.2
QL4	112°18′39.888″	16°56′31.472″	45.4	QH24	112°11′27.665″	16°58′54.056″	97.6
QL5	112°19′31.573″	16°56′23.678″	16.5	QH25	112°11′18.943″	16°58′09.164″	90.3
QL6	112°20′48.502″	16°57′04.015″	39.9	IL26	112°13′11.684″	16°45′18.651″	33.2
QL7	112°19′25.512″	16°55′45.652″	45.5	IL27	112°12′59.184″	16°46′37.231″	16.7
QL8	112°20′23.188″	16°54′25.495″	20.1	IL28	112°12′53.783″	16°47′39.007″	31.1
QM9	112°21′30.552″	16°56′14.587″	59.2	IL29	112°13′47.543″	16°47′22.948″	49.1
QM10	112°21′37.774″	16°55′10.191″	59.8	IL30	112°15′21.089″	16°45′50.219″	43.3
QM11	112°21′02.837″	16°53′47.788″	63.4	IL31	112°15′22.167″	16°44′17.336″	48.1
QM12	112°20′37.648″	16°53′06.029″	60.1	IM32	112°16′58.167″	16°44′19.249″	59.7
QM13	112°20′13.097″	16°53′29.095″	55.6	IH33	112°12′20.320″	16°45′52.874″	88.2
QM14	112°19′25.625″	16°54′14.509″	51.1	IH34	112°17′52.638″	16°44′29.063″	80.8
QM15	112°17′49.744″	16°54′15.635″	57.1	YL35	112°22′31.561″	16°46′33.159″	34.1
QM16	112°16′13.693″	16°54′16.471″	58.3	YM36	112°20′56.833″	16°46′33.766″	52.1
QM17	112°14′43.911″	16°53′05.624″	63.6	YM37	112°19′20.472″	16°45′2.555″	56.5
QM18	112°14′37.603″	16°54′17.186″	68.8	YM38	112°20′9.039″	16°45′48.223″	59.1
QM19	112°14′39.385″	16°55′48.354″	63.1	YM39	112°19′22.209″	16°46′33.138″	59.6
QM20	112°14′39.003″	16°57′21.742″	53.4				

**TABLE 2:** Samples from different islands.

Island	Samples
Qilian Island	QL1, QL2, QL3, QL4, QL5, QL6, QL7, QL8, QM9, QM10, QM11, QM12, QM13, QM14, QM15, QM16, QM17, QM18, QM19, QM20, QM21, QH22, QH23, QH24, QH25
Iltis Bank	IL26, IL27, IL28, IL29, IL30, IL31, IM32, IH33, IH34
Yongxing Island	YL35, YM36, YM37, YM38, YM39

**TABLE 3:** Samples from different depths.

Depth	Samples
L (15–50 m)	QL1, QL2, QL3, QL4, QL5, QL6, QL7, QL8, IL26, IL27, IL28, IL29, IL30, IL31, YL35
M (50–70 m)	QM9, QM10, QM11, QM12, QM13, QM14, QM15, QM16, QM17, QM18, QM19, QM20, QM21, IM32, YM36, YM37, YM38, YM39
H (70–100 m)	QH22, QH23, QH24, QH25, IH33, IH34

### Measurement of Environmental Factors

The sediment samples were centrifuged at 5000 rpm for 30 min at room temperature, and the supernatant sediment pore water was filtered through a 0.45 μm Millipore filter. The pH and salinity of the sediment pore water were measured using a YSI Professional Plus handheld multi-parameter water quality meter (Yellow Springs Instrument Company, United States). The concentrations of ammonium (NH_4_^+^), nitrate (NO_3_^–^), and nitrite (NO_2_^–^) were measured by using UV-visible spectrophotometer HP 8453 (Hewlett-Packard, United States) at wavelengths of 220–275, 540, and 420 nm, respectively. Ion chromatography was employed for the detection of chloridion (Cl^–^), sulfate (SO_4_^2–^), calcium (Ca^2+^), and potassium (K^+^) (Thermo Fisher, United States). The total organic carbon (TOC) of each sample was determined by a TOC analyzer (Shimadzu, Japan) with a detection limit of 0.1 mg/L. Sediment samples were processed as previously described (Shuqing et al., 2010), and sediment particle size was measured using a Mastersizer 2000 laser particle size analyzer (Malvern Instruments, United Kingdom).

### 16S Amplicon Sequencing and Data Processing

Total DNA from each sample (0.5 g) was extracted using a PowerSoil DNA Extraction Kit (QIAGEN Instruments, United States). The extracted DNA was stored at −20°C for further use and at −80°C for permanent preservation. The quantity and quality of isolated DNA were evaluated using a Nano Drop spectrophotometer (Thermo Fisher Scientific, United States) and agarose gel electrophoresis (Bio-Rad, United States), respectively.

The V4-V5 regions of the bacterial 16S ribosomal RNA gene was amplified by PCR (95°C for 2 min, followed by 25 cycles of 95°C for 30 s, 55°C for 30 s, and 72°C for 30 s and then final extension at 72°C for 5 min) using the 515F 5′-barcode-GTGCCAGCMGCCGCGG)-3′ and 907R 5′-barcode-CCGTCAATTCMTTTR AGTTT-3′ primers, where the barcode was an eight-base sequence unique to each sample. PCR reactions were performed in triplicate 20 μL mixtures containing 4 μL of 5× FastPfu Buffer, 2 μL of 2.5 mM dNTPs, 0.8 μL of each primer (5 μM), 0.4 μL of FastPfu Polymerase, and 10 ng of template DNA. Amplicons were extracted from 2% agarose gels and purified using an AxyPrep DNA Gel Extraction Kit (Axygen Biosciences, United States), according to the manufacturer’s instructions, then quantified using QuantiFluor^TM^-ST (Promega, United States). Purified amplicons were pooled in equimolar concentrations and paired-end sequenced (2 × 300) on an Illumina MiSeq platform according to the standard protocols.

Raw fastq files were demultiplexed, then quality-filtered using QIIME (version 1.17) with the following criteria: (i) the 300 bp reads were truncated at any site receiving an average quality score <20 over a 50 bp sliding window and truncated reads that were shorter than 50 bp were discarded; (ii) exact barcode matching, two nucleotide mismatches during primer matching, reads containing ambiguous characters were removed, and (iii) only sequences that overlapped more than 10 bp were assembled according to their overlap sequence. Reads that could not be assembled were discarded.

SILVA 132 rRNA database^1^ was employed as the reference database, and RDP Classifier 2.11 was used for taxonomy annotation^2^. Operational Taxonomic Units (OTUs) were clustered with a 97% similarity cutoff using UPARSE (version 7.1)^3^ and chimeric sequences were identified and removed using UCHIME.

### Statistical Analysis

Among the 39 samples, the particles of 34 sediment samples were in compliance with the Mastersizer 2000 test. The Udden-Wentworth φ particle size standard was used for the grain size, and the average particle size was calculated by the moment method (McManus, 1988) as follows: Average particle size (Mz) = 1/φ. The remaining five samples, QL6, QL8, QM13, QM14, and SL35, could not be measured because of the large particle size and were therefore assigned values of 0.331, 0.332, 0.333, 0.334, and 0.335, respectively, based on three times the standard deviation.

To determine the alpha diversity, we rarified the OTUs and calculated the Chao index (species abundance), Shannon’s diversity (community diversity), and Shannon’s evenness (community uniformity). To test whether the sequencing results represented the actual situation of the microorganisms in the sample, the Coverage value reflecting the coverage of the sample library was calculated using Mothur. Bray-Curtis dissimilarity between samples was calculated using the R package ecodist. In addition, Spearman’s correlation between environmental factors and microbial diversity indices were calculated using the R package psych. Wilcoxon rank-sum tests of environmental factors among different islands or depths were conducted using the R package stats. Heatmaps to show the composition of the bacterial community and Spearman’s correlation between bacterial abundance and environmental factors were generated using the R package psych. Differential testing of bacterial abundance at the genus level among islands or depth groups was conducted using the Kruskal-Wallis H test in the R package statistics, and the *p*-value was checked using false discovery rate (FDR).

To conduct co-occurrence network analysis, the Spearman correlations of bacteria were computed based on the relative abundance of genus, and the networks were visualized using the R igraph package,^4^ after which the modularity of the network was calculated and modules were detected using the greedy modularity optimization method (Deng et al., 2012). Non-metric multidimensional scaling (NMDS) analysis based on Bray-Curtis distance was performed using the R package “ecodist,” which demonstrated the similarity of the microbial community structure among the 39 samples. To analyze the similarities among samples from different depths and spatial distribution, ANOSIM was employed to examine whether the differences among the groups were significantly higher than those within groups, which was calculated using the R package vegan, function “anosim.” Constrained ordination methods (CCA) were calculated using the R package vegan, function “cca” to investigate the key factors affecting the variation in the microbial communities among different sites.

## Results and Discussion

### Physicochemical Factors of Sediments

The particle size distribution curve of the sediments showed two clear components in the grain-size frequency curves (Supplementary Figure S1). The finer peaks concentrate at ca. 30 μm, which can be found for all the samples except QM12 (Supplementary Figure S1A) and IL27 (Supplementary Figure S1C), containing one peak at ∼60 μm. On the contrary, the coarser peaks distribute at ca. 120 μm, existing only in several samples either depositing in proximal or at the steep slope, and all the samples are at Qilian Island except IH34. The finer peak likely represents the mean hydro-energy of the current westward in our study area, while the appearance of the coarse fraction is determined by the submarine topography, location or the source distance. The mean grain-size of the samples collected from the open water area is relatively small and characterized by the solely finer component, might be indicative of the influence of the current transportation. However, as a consequence of proximal deposition, sediments collected from the areas close to the islands and banks are significantly coarser, such as sample QM10 and QM11. Moreover, as representative of the maximum hydro-energy, cumulative 1% of the grain-size for these coarse samples around 1900 μm correlated to the rolling transportation (Passega, 1957, 1964). Therefore, we inferred that the commonly existing fine fraction with peak at 30 μm was indicative of the current hydro-energy, and the coarse fraction is related to the proximal deposition especially in Qilian Island.

The environmental factors are shown in Supplementary Table S1. The concentrations of TOC of pore water of sediments at Qilian Island were significantly higher than those at Iltis Bank (Wilcoxon’s test, *p* < 0.01), suggesting that the content of organic matter in the sediment of Qilian Island was significantly higher than that at Iltis Bank. The concentrations of NH_4_^+^ and PO_4_^3–^ at Qilian Island were significantly higher than those at Yongxing Island (Wilcoxon’s test, *p* < 0.01). The concentrations of TOC in sediments of water depth H (70–100 m) were significantly higher than those of water depth M (50–70 m) (Wilcoxon’s test, *p* < 0.05). The concentrations of NO_2_^–^ in sediments of water depth M (50–70 m) were significantly higher than those of water depth L (15–50 m) (Wilcoxon’s test, *p* < 0.05). The concentrations of NO_3_^–^ in sediments of water depth H (70–100 m) were significantly higher than those of water depth L (15–50 m) (Wilcoxon’s test, *p* < 0.05), suggesting that the concentrations of TOC and NO_3_^–^ in sediments of water depth H (70–100 m) were higher than those of water depth 15–70 m.

### Microbial Community Structure and Its Correlation With Environmental Factors

A total of 4,404,200 high-quality sequences were retained for post-run analysis. A total of 9698 OTUs were assigned at a 3% dissimilarity threshold. The microbial diversity indices are shown in Supplementary Table S2. The correlation between the microbial diversity index and environmental factors showed that the Shannon and Shannon even indices were significantly negatively correlated with TOC, NO_3_^–^, and depth, while Chao index was significantly negatively correlated with TOC and NO_3_^–^ (Table 4).

**TABLE 4:** Correlations between environmental factors and microbial diversity indices.

	Shannon	Chao	Shannoneven
Depth	−0.338*	–0.191	−0.371*
TOC	−0.671***	−0.587***	−0.650***
NH_4_^+^	–0.268	–0.261	–0.307
PO_4_^3–^	–0.276	–0.176	−0.320*
NO_2_^–^	–0.302	–0.182	–0.302
NO_3_^–^	−0.686***	−0.520***	−0.698***
Cl^–^	–0.171	–0.050	–0.169
SO_4_^2–^	–0.033	0.120	–0.060
K^+^	0.114	0.156	0.116
Ca^2+^	–0.027	0.113	–0.030
Mz	–0.110	0.013	–0.114
Salinity	0.228	0.121	0.261
pH	0.271	0.270	0.295

Spearman correlation: *, p < 0.05; ***, p < 0.001.

**FIGURE 2:**
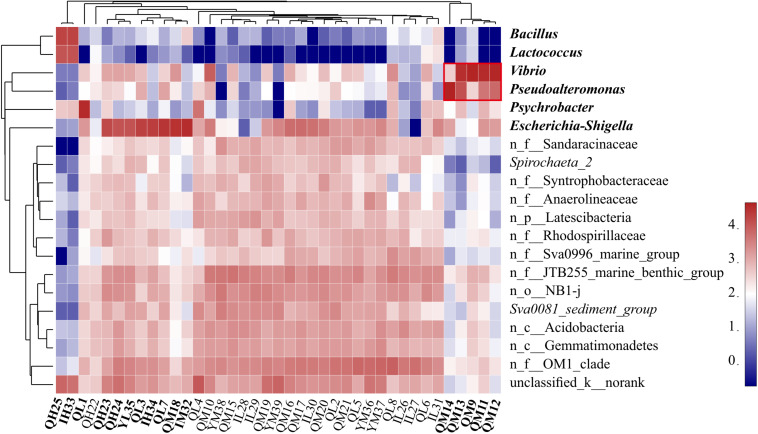
Heatmap of the relative abundance of the top 20 genera of all sediment samples. Clusters based on microbial community composition are above the heatmap, clusters based on the genera are on the left of the heatmap, and special samples and bacteria are shown in bold. n_ indicates no rank. The data were scaled per column. The values represent the relative abundance of genera in the sample.

The dominant phyla in the sediments were *Proteobacteria* (55.50%, mainly γ-, δ-, α-), *Actinobacteria* (8.54%), *Bacteroidetes* (6.56%), and *Firmicutes* (6.27%) (Supplementary Figure S2). *Proteobacteria* are widely found in sediments and distributed in marine sediments worldwide (Bienhold et al., 2016). The top 20 dominant genera were *Escherichia-Shigella*, *Vibrio, Pseudoalteromonas, Psychrobacter, Bacillus*, and *Lactococcus*, and some norank bacteria (Figure 2). We found that *Bacillus* and *Lactococcus* had a high abundance in samples QH25 and YH33. Most *Bacillus* are saprophytic, with a wide range of habitats, and some strains are pathogenic (Ruger et al., 2000). *Lactococcus* is found in some cultured marine fish and can cause fish diseases (Tanekhy, 2013). *Psychrobacter* was in high abundance in sample QL1, and was detected only in oil-contaminated waters (Prabagaran et al., 2007). Additionally, *Escherichia-Shigella* was found in high abundance at eight sampling points from QH23 to YM32 distributed at different water depths and different islands (Figure 2). *Escherichia-Shigella* is a pollution indicator and part of the main bacterial group responsible for diarrhea worldwide (Brunette et al., 2015). We speculate that the frequent and complex horizontal and vertical currents in the SCS (Liu and Gan, 2017) and the frequent flow of people in this region are likely responsible for the presence and spread of *Escherichia-Shigella*. *Vibrio* was found to be highly abundant at four sampling points (QM9, QM11, QM12, and QM13). Many strains of *Vibrio* are well-known pathogenic bacteria that can cause disease in humans or marine animals (Xiaohua et al., 2018). The increased abundance of *Vibrio* is associated with algal outbreaks (such as diatoms and brown algae) (Miller et al., 2005; Gilbert et al., 2012). In the Deepwater Horizon oil spill in the Gulf of Mexico, *Vibrio* was dominant in the petroleum-associated microbial community in surface seawater samples (Hamdan and Fulmer, 2011) and salt marsh plant samples contaminated by petroleum foam (Liu and Liu, 2013). In addition, QM9, QM11, QM12, and QM13 at southeast of Qilian Island are exposed to frequent human activities. Therefore, it is very likely that the domestic garbage on the island could lead to eutrophication of the sea, causing high growth of *Vibrio*. *Pseudoalteromonas* was highly abundant in QM14, reaching 93.32%. This genus is mainly distributed in open sea and coastal waters, affecting sedimentation, germination, and metamorphosis of various invertebrates and algae (Holmstrom and Kjelleberg, 1999; Bowman, 2007). In the present study, high abundances of *Escherichia-Shigella, Vibrio, Pseudoalteromonas, Bacillus, Psychrobacter*, and *Lactococcus* were distributed almost around Qilian Island, suggesting that Qilian Island has rapidly developed with frequent human activities, including garbage disposal, farming, and oil spills from ships, which had caused an increase in pathogenic bacteria and pollution indicator bacteria.

**FIGURE 3:**
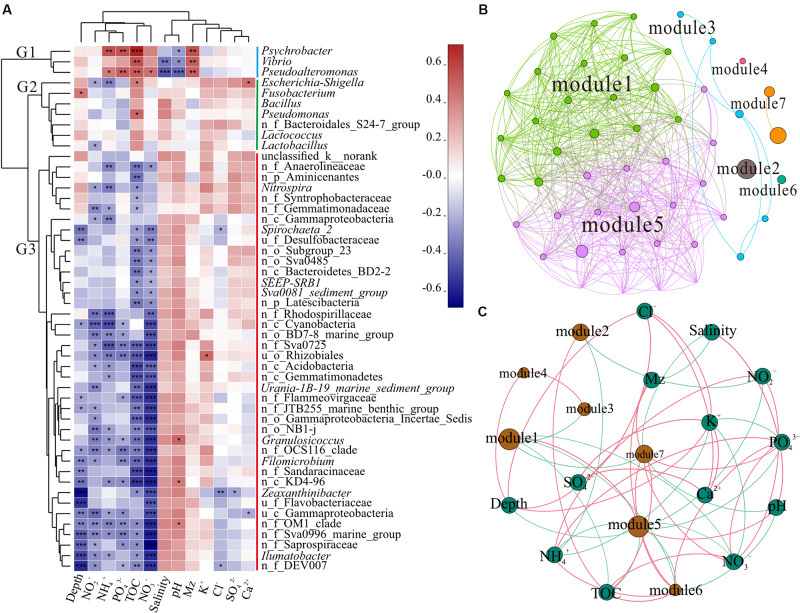
Correlation analysis. **(A)** Correlation heatmap between the top 50 genera and environmental factors. Clusters based on environmental factors are above the heatmap and clusters of genera are on the left of the heatmap; n_ and u_ indicate no rank and unclassified, respectively. The asterisks in the cell represent significant correlation, *p < 0.05, **p < 0.01, and ***p < 0.001. The values denote correlation coefficient. **(B)** Network of the top 50 genera. Top 50 abundant genera in all 39 samples were selected to calculate the correlation among bacteria. Each node represents a genus, the nodes are colored by module, and the connection between the nodes represents a significant correlation (Spearman, *p* < 0.05, *r* > 0.6). **(C)** Network of the correlation between modules and environmental factors (Spearman, *p* < 0.05, *r* > 0.3) across all 39 samples. The sum of all bacterial abundances in the module represents the richness of the module. Brown nodes represent the modules of the microbiome, and the sizes of the brown nodes indicate the relative abundance of the bacteria. Green nodes represent environmental factors. Red edge represents positive correlation and green edge denotes negative correlation.

As shown in Figure 3A, the top 50 genera were divided into three clusters, G1, G2, and G3, based on the clustering tree. G1 was positively correlated with NH_4_^+^, PO_4_^3–^, TOC, and Mz, but was negatively correlated with salinity and pH. However, G2 was positively correlated with TOC. G3 contained the largest number of genera, almost all of which were unnamed bacteria in the ocean and negatively correlated with depth, NO_2_^–^, NH_4_^+^, PO_4_^3–^, TOC, and NO_3_^–^. The CCA further revealed the correlation between environmental factors and unique genera (Supplementary Figure S3). The genera *Vibrio* and *Pseudoalteromonas* clustered in G1 had positive correlation with Mz, PO_4_^3–^, NO_3_^–^, and TOC; *Psychrobacter* was mainly affected by NH_4_^+^ and TOC; and *Escherichia-Shigella*, *Bacillus*, *Pseudomonas*, and *Lactococcus* belonging to G2 presented positive relationship with TOC. These findings indicated that different taxon clusters in the marine sediment showed varied responses to environmental factors. However, the effects of different bacteria and functional groups on environmental factors need to be further investigated, along with the influence of environmental factors on different bacteria and functional groups.

In complex environments, network analysis provides a promising beginning for exploration of the organization and dynamics of microbial interactions and niches (Duran-Pinedo et al., 2011; Zhou et al., 2011; Faust and Raes, 2012). To identify functional groups that may interact with each other or share niches in sediments, we constructed a co-occurrence network of sediment bacteria in the study area. Upon modular analysis of the created network, 50 nodes and 468 edges were divided into seven modules (top 50 genera, *r* > 0.6, *p* < 0.05, Supplementary Table S3). There were many unclassified and norank bacteria in modules 1 and 5, and they were widely distributed and dominant in the marine environment (Figure 3B). These findings suggest that modules 1 and 5 have high co-occurrence, which may represent two main co-evolutions of functional communities. To investigate the factors affecting the classification and abundance of bacterial clusters, we analyzed the network diagram of the correlation between modules and environmental factors (Figure 3C). Modules 1 and 5, which had high diversity, shared positive relationship with each other, but had negative relationship with modules 3, 6, and 7. Unlike modules 1 and 5, modules 2, 6, and 7 were positively correlated with TOC. Modules 6 and 7 were positively correlated with Mz.

Polluted areas usually show higher types of pollutant resistance and different community compositions, when compared with pristine areas (Sun et al., 2013; Quero et al., 2015; Gubelit et al., 2016). The presence of potential pathogenicity and pollution indicator bacteria, such as *Vibrio*, *Pseudoalteromonas*, *Escherichia-Shigella, Psychrobacter*, and *Pseudomonas*, along with strong hydrodynamic conditions of the current migrating westward, makes the ecological risks more serious. Different modules reflect the habitat heterogeneity, which is affected by different environmental selection mechanisms (Thompson, 2005; Olesen et al., 2007). In addition, modules 3 and 7 (*Vibrio*, *Pseudoalteromonas*, *Lactococcus, Bacillus*, and *Pseudomonas*) showed negative correlation with modules 1 and 5 (dominant microbial functional groups), indicating that the increase in pathogenic and pollution indicator bacteria may decrease the diversity of pristine bacteria in the SCS. Thus, sediment microbial communities of the SCS disturbed by human activities could present altered interactions of bacteria and correlation between functional groups, which might affect the bacterial element cycle and ecological function in sediment.

### Spatial Distribution of Microbial Community in Xuande Atoll Sediments

**FIGURE 4:**
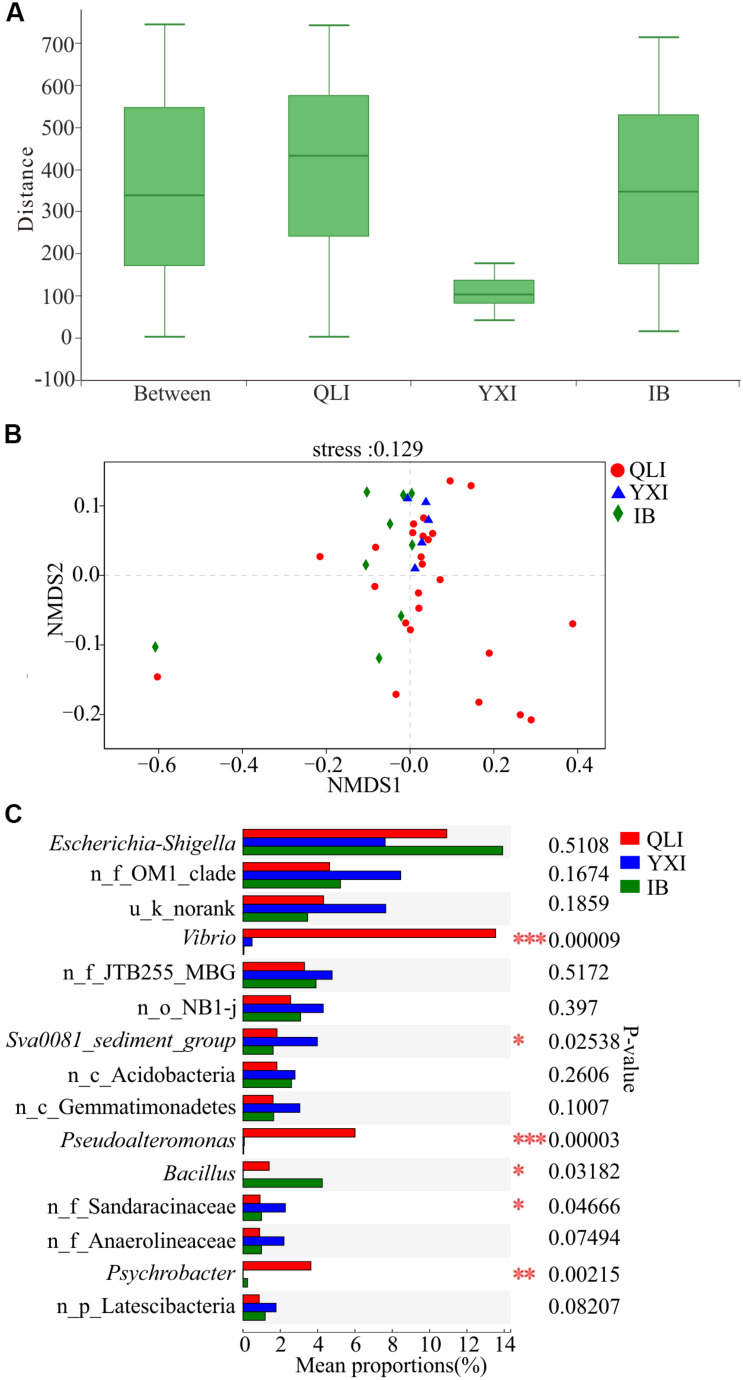
Difference in microbial communities among three islands. QLI, YXI, and IB represent Qilian Island, Yongxing Island, and Iltis Bank, respectively. **(A)** Similarity within and between islands. ANOSIM analysis based on Bray-Curtis distance of bacterial community structure at the OTU level (*p* = 0.775, *r* = –0.0785). **(B)** NMDS analysis based on Bray-Curtis distance of bacterial community structure at the OTU level among all 39 sediment samples. **(C)** Differences in bacterial abundance among three islands based on the Kruskal-Wallis test, and the *p*-value was checked using FDR. *p < 0.05; **p < 0.01; ***p < 0.001. n_ and u_ indicate no rank and unclassified, respectively, MBG represents marine_benthic_group.

There were no significant differences in the richness, diversity, and evenness of microbial communities among the three islands (Wilcoxon test, *p* > 0.05, Supplementary Table S2). Previous studies have shown that geographical location has a strong impact on the composition of microbial communities, and the β diversity distance attenuation model of bacterial community composition depends on the spatial scale (Martiny et al., 2011; Zinger et al., 2011). A study of the SCS showed that control of the environment and diffusion limiting microorganisms will result in a distribution pattern of biogeography (Zhang et al., 2014). Through ANOSIM analysis of bacterial community composition between the three islands at the OTU level (Figure 4A), the within-group difference of Qilian Island was greater than the difference between the islands, while that of Yongxing Island was the lowest. For Iltis Bank, the within-group difference was relatively large and slightly lower than the difference between the groups. The results of NMDS analysis also showed that the sediment samples around Qilian Island were most dispersed, indicating that the microbial community structure near Qilian Island was most diverse (Figure 4B). The sample points at Yongxing Island were closest together. The sample points at Iltis Bank were also scattered.

Subsequently, the microbial community structures of three islands were compared at the genus level (Figure 4C). The abundance of *Vibrio*, *Pseudoalteromonas*, and *Psychrobacter* at the three islands was significantly different, and the abundance of these three genera in Qilian Island was higher than that in Yongxing Island and Iltis Bank. In Qilian Island, *Vibrio* was abundant in sample QM9 (84.6%), QM11 (73.3%), QM12 (80.3%), and QM13 (66.8%); *Pseudoalteromonas* was abundant in sample QM11 (9.1%), QM12 (12.5%), QM13 (25.8%), and QM14 (93.3%); and *Psychrobacte*r was abundant in sample QL1 (74.3%). These findings suggested that the differences in community structure were mainly between unique sites (QM1, QM9, QM11, QM12, QM13, QM14) and the genera *Psychrobacte*r, *Vibrio*, and *Pseudoalteromonas*.

### Effects of Different Depths on Microbial Community Structure in Sediments

The depth of water significantly affects the community structure of microorganisms in coastal areas (Hewson et al., 2007; Boer et al., 2009). For instance, sediment depth related patterns in bacterial community structure were detected in a variety of benthic habitats, such as cold seep sediments (Inagaki et al., 2002), warm deep Mediterranean sea (Luna et al., 2004), coral reef sediments (Hewson and Fuhrman, 2006), as well as continental shelf sediments of the southern North Sea (Franco et al., 2007). Environmental parameters, such as wave impacts (Hewson and Fuhrman, 2006), organic carbon and chlorophyll *a* contents in the sediment (Polymenakou et al., 2005), eutrophication associated with fish farms (Vezzulli et al., 2002), and inorganic nutrients enrichment (Hewson et al., 2003), have been found to be related to shifts in the benthic bacterial community structure. In this study, the differences in Wilcoxon rank-sum test of microbial diversity indices in the sediments from different water depths showed that the Shannon indices of L (15–50 m) and H (70–100 m) groups were significantly different (Wilcoxon test, *p* < 0.05, Supplementary Table S2). In addition, Chao index of M (50–70 m) and H (70–100 m) groups were significantly different (Wilcoxon test, *p* < 0.05, Supplementary Table S2). These findings indicated a decreased microbial diversity along the vertical depth in the ocean.

**FIGURE 5:**
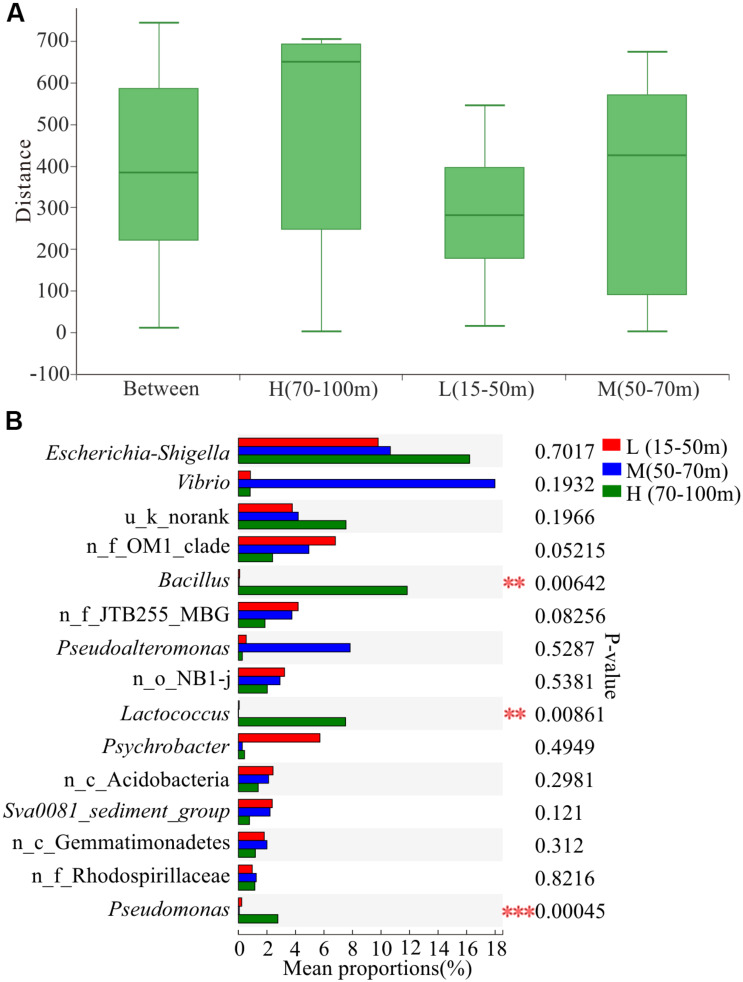
Variation in microbial communities among three different depths. L, M, and H represent samples from depths of 15–50, 50–70, and 70–100 m, respectively. **(A)** Similarity analysis within and between depths. ANOSIM analysis based on Bray-Curtis distance of bacterial community structure at the OTU level (*p* = 0.004, *r* = 0.1911). **(B)** Differences in bacterial abundance among three depths based on the Kruskal-Wallis test and the p-value was checked using FDR. **p < 0.01; ***p < 0.001. n_ and u_ indicate no rank and unclassified, respectively; MBG represents marine_benthic_group.

The results of ANOSIM at the OTU level (Figure 5A) showed that the within-group difference of water depth L (15–50 m) was less than the difference between the groups, indicating that the microbial community structure in the water depth L (15–50 m) was similar. In addition, the sample points of water depth M (50–70 m) were scattered, and the within-group difference was slightly larger than the difference between the groups. Moreover, the within-group difference of water depth H (70–100 m) was significantly larger than the difference between groups. The microbial community structure at water depth H (70–100 m) presented the most significant difference. The proportion of *Vibrio* at water depth M (50–70 m) was the highest, reaching 18.54% (Figure 5B). In addition, the proportion of *Bacillus* differed significantly among the three water depths, with the highest level of 12.20% being observed at water depth H (70–100 m). Furthermore, the proportion of *Pseudoalteromonas* at water depth M (50–70 m) was 8.07%. Overall, the proportion of pathogenic and pollutant metabolism associated bacteria at the depth of 50–100 m was significantly higher than that in the shallow water layer of 15–50 m across all the 39 samples, indicating that the study area had been contaminated and the deep water layer is more severely polluted than the shallow water layer.

**FIGURE 6:**
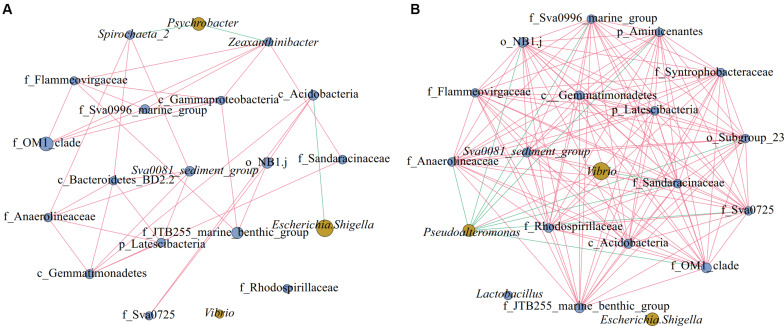
Networks of microorganisms in sediment of different water depths. Co-occurrence of the top 20 genera in sediment of different water depths: **(A)** Depth L (15–50 m), **(B)** Depth M (50–70 m). Network diagrams were constructed using 15 (L) and 15 (M) samples; in the group M (50–70 m), we discarded the samples QM17, QM20, and SM36. Blue nodes represent indigenous bacteria and yellow nodes denote pathogenic and pollutants-degrading bacteria. The size of the nodes represents the relative abundance of the bacteria. The edge between the points represents a significant correlation, with the red edge signifying positive correlation and the green edge denoting a negative correlation (Spearman, *p* < 0.01, *r* > 0.6).

As shown in Figure 6, there were 20 nodes in each of the two network diagrams (A and B; top 20 genera, *p* < 0.01, *r* > 0.6), and there were 35 and 131 edges in the sediment of water depths L (15–50 m) and M (50–70 m), respectively. In addition, there were 32 and 121 edges with positive correlations and 3 and 10 edges with negative correlations, respectively. Moreover, the number of connections in the network increased from the sediment of water depth L (15–50 m) to M (50–70 m), and the proportion of positive correlation edges at the sediment of water depth L (15–50 m) and M (15–50 m) were 91.4 and 92.4%, respectively. Many positive interactions have been found to occur in natural bacterial populations (Morris et al., 2012; Hallam and McCutcheon, 2015). The network represents coordinated variability, where a common change in abundance of members reflects interactions between members or responds to environmental factors (Shi et al., 2016). Studies on macrobiology and microbiology have shown that resources and food supply are important drivers of ecological network structure (Henzi et al., 2009; Foster et al., 2012). In the sediment of water depth L (15–50 m), *Psychrobacter* was negatively correlated with *Spirochaeta_2* and *Zeaxanthinibacter*, and *Escherichia-Shigella* was negatively correlated with c_Acidobacteria (Figure 6A). In the sediment of water depth M (50–70 m), *Pseudoalteromonas* was negatively correlated with other bacteria except *Vibrio*, and *Vibrio* was negatively correlated with f_Sandaracinaceae (Figure 6B). Besides, another network diagram was constructed based on 39 samples (Supplementary Figure S4) using Spearman correlation with *p* < 0.01 and *r* > 0.5, and it was found that *Psychrobacter* was negatively correlated with f_Sandaracinaceae and f_Anaerolineaceae, and *Lactococcus* was negatively correlated with *Sva0081_sediment_group*, but positively correlated with *Bacillus*. There was a significant positive correlation among all indigenous bacteria. *Psychrobacter* and *Lactococcus* are pollutants-degrading bacteria, suggesting that the indigenous bacterial group may have been affected. Therefore, we speculate that, in our study area, the relationship between microbes shows negative correlation between pathogenic and pollutants-degrading bacteria and indigenous bacteria, and that this phenomenon may be affected by pollution and competition among different ecological groups. When the seawater is polluted, the ecological group may change more severely, and the competition between the pathogen and indigenous group would make recovery of the ecological environment difficult.

## Conclusion

In the Qilian Island area, high abundance of potential pathogenic bacteria, *Escherichia-Shigella*, *Lactococcus*, and *Pseudoalteromonas*, and pollutants-degrading bacteria, *Bacillus* and *Vibrio*, revealed that this study area is likely to have been contaminated, and that the pollution is mainly affected by geographical location and hydrodynamic conditions, with a possible tendency to spread. The existence of unique genera in the study area is the main reason for the difference in community structure between islands. The pathogenic bacterial group was negatively correlated with most of the indigenous bacteria in the ocean, suggesting that the indigenous bacterial group might have been influenced, and that the complexity of this effect is reflected in how different groups of microbes respond to different environmental factors, the pattern of interaction between ecological groups, and mechanisms of microbial community formation at different depths. Because of the increased human activity in the area, the impact of anthropogenic activities on microbial community structure and the potential changes in the role of microorganisms in the cycle of elements require further research.

## Data Availability Statement

The accession number for sequencing data in NCBI is SRP222491.

## Author Contributions

BZ did the writing and data analysis of this research. YY and S-ZX participated in the experiment of samples used in this study. YL, RY, and M-PL participated in the data analysis and discussion of this research. X-MW and Y-LX did the collection of the samples for this study. X-YG participated in the topic and discussion of the whole study.

## Conflict of Interest

The authors declare that the research was conducted in the absence of any commercial or financial relationships that could be construed as a potential conflict of interest.

## Footnotes

1 https://www.arb-silva.de/

2 http://sourceforge.net/projects/rdp-classifier/

3 http://drive5.com/uparse/

4 http://igraph.org

## Supplementary Material

FIGURE S1 Particle size distribution curve of sediment samples.

FIGURE S2 Proportion of bacteria at the phylum level. u_ indicates unclassified.

FIGURE S3 CCA analysis of microbial and environmental factors.

FIGURE S4 Network of microorganisms based on 39 samples.

TABLE S1 Environmental variables of all sediment samples.

TABLE S2 Biodiversity indices of bacterial communities.

TABLE S3 Genera in different modules from network analysis.
