# Supplementary figures and images for: Sediment Microbial Communities and Their Potential Role as Environmental Pollution Indicators in Xuande Atoll, South China Sea
Source: Front Microbiol. 2020 May 25;11:1011. doi: 10.3389/fmicb.2020.01011 (PMC7261833; doi:10.3389/fmicb.2020.01011)

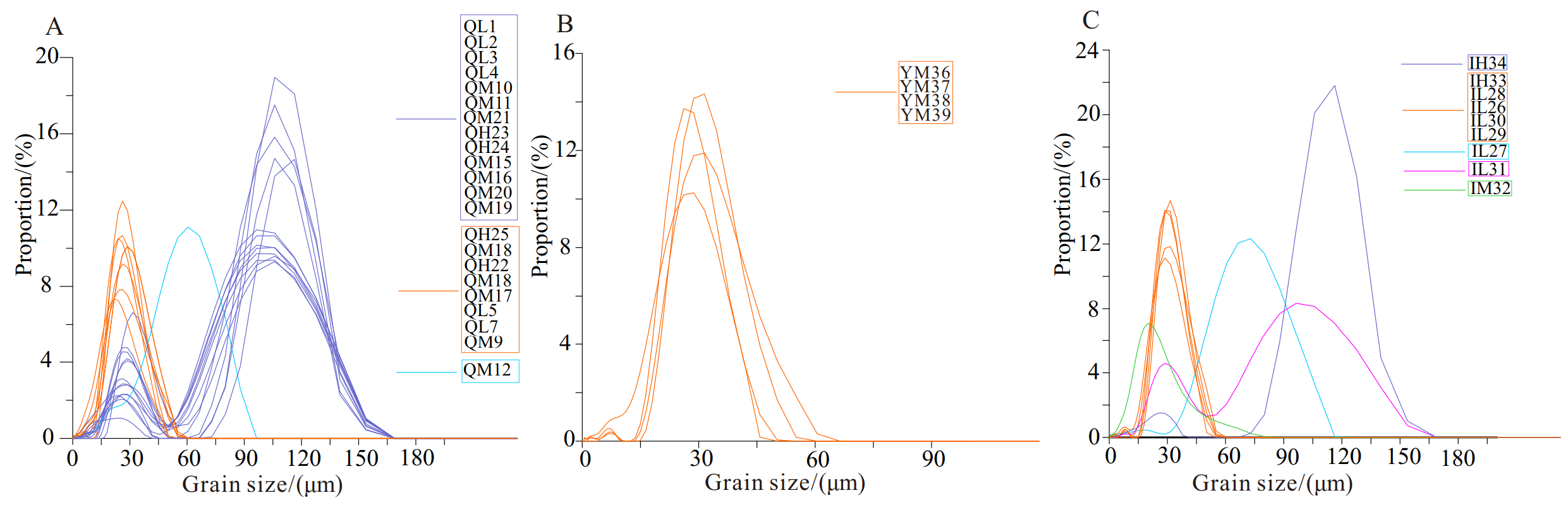

Supplement: FIGURE S1 — Particle size distribution curve of sediment samples. [file Image_1.TIF]

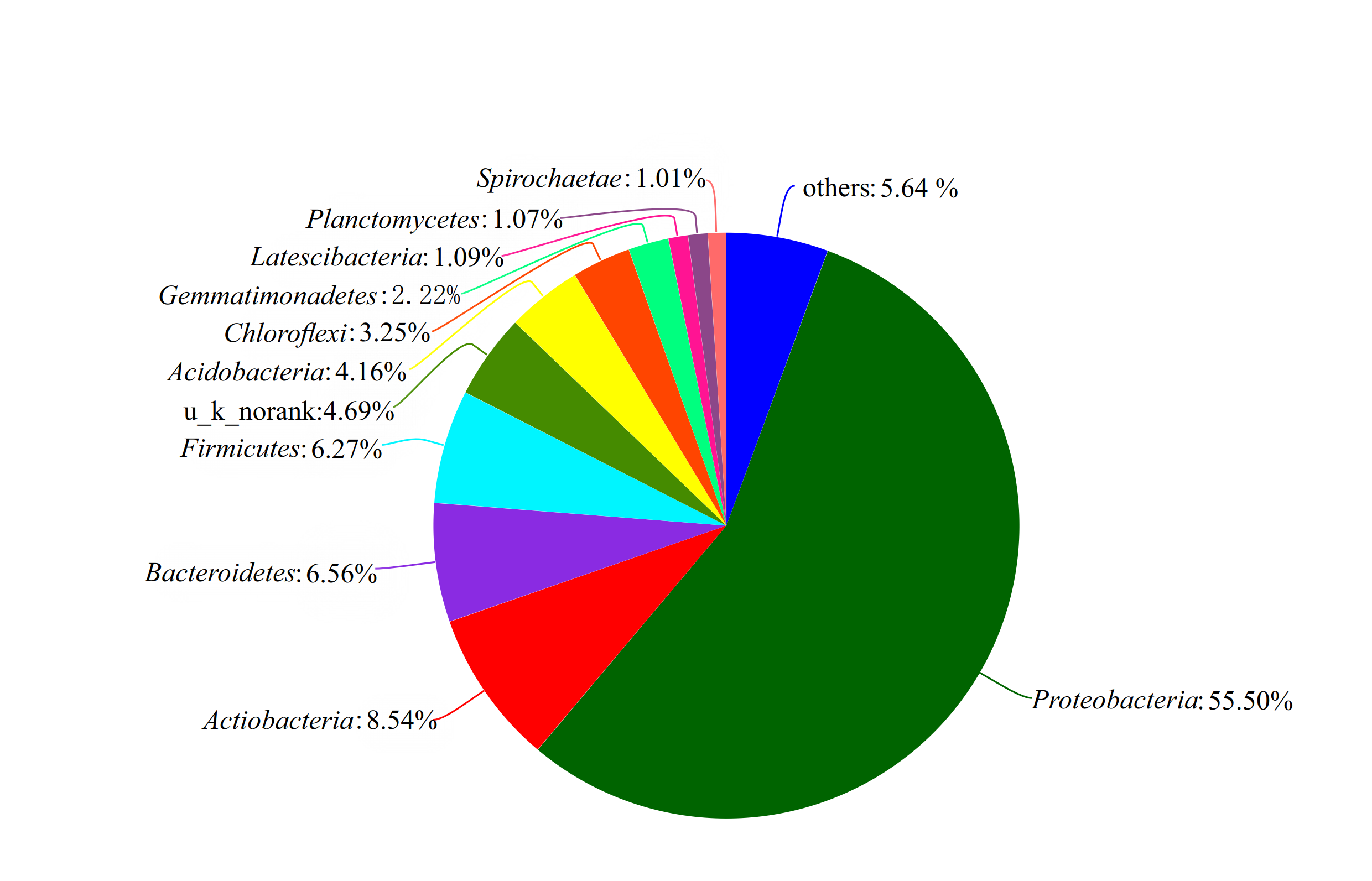

Supplement: FIGURE S2 — Proportion of bacteria at the phylum level. u_ indicates unclassified. [file Image_2.TIF]

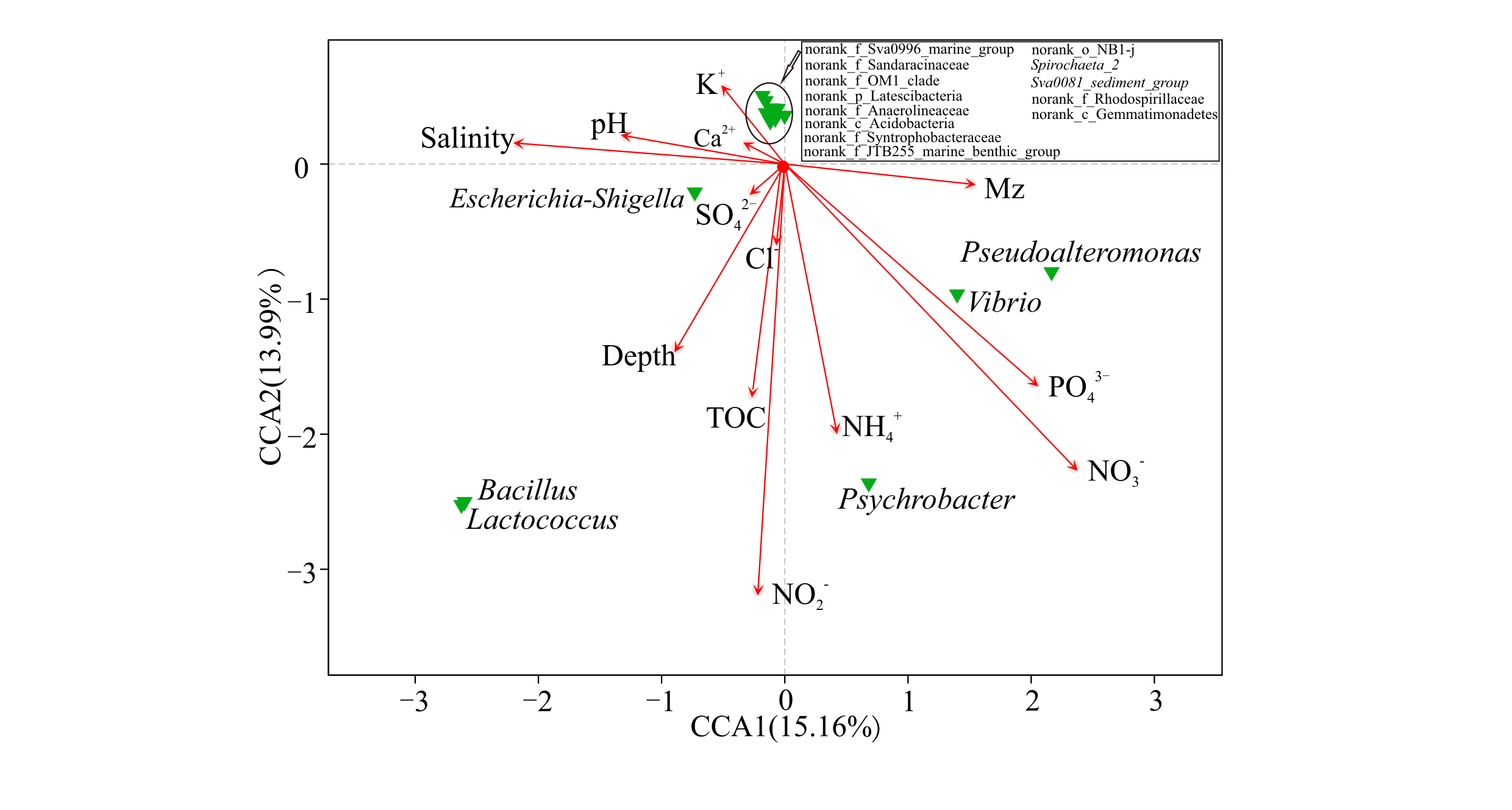

Supplement: FIGURE S3 — CCA analysis of microbial and environmental factors. [file Image_3.TIF]

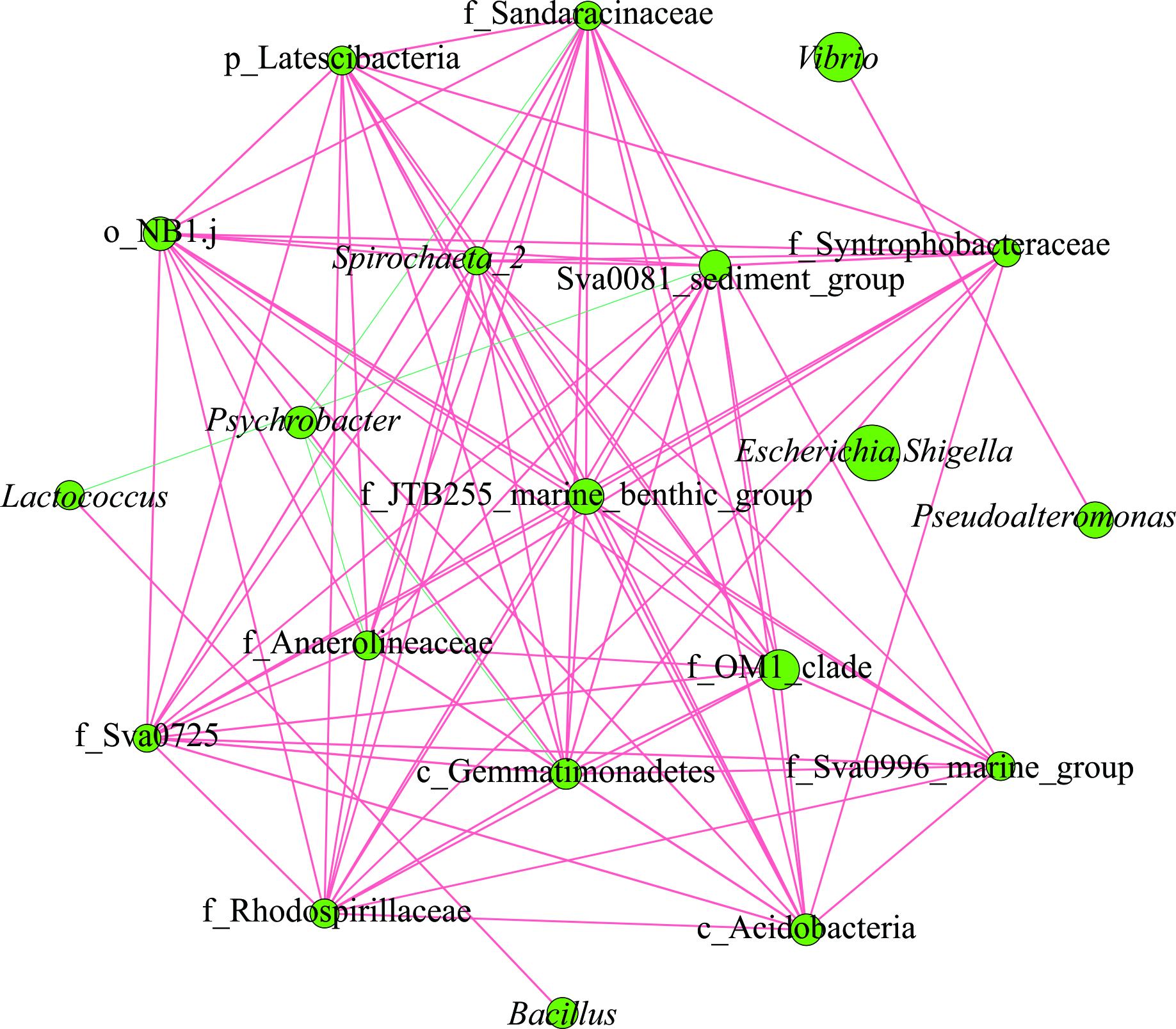

Supplement: FIGURE S4 — Network of microorganisms based on 39 samples. [file Image_4.TIF]
