# Supplementary material for: Sediment Microbial Communities and Their Potential Role as Environmental Pollution Indicators in Xuande Atoll, South China Sea
Source: Front Microbiol. 2020 May 25;11:1011. doi: 10.3389/fmicb.2020.01011 (PMC7261833; doi:10.3389/fmicb.2020.01011)
Supplement: TABLE S1 — Environmental variables of all sediment samples. [file Data_Sheet_1.docx]

**Table S1.** Environmental variables of all sediment samples.

| **station** | **TOC (mg/L)** | **NH_4_^+^ (mg/L)** | **NO_3_^-^ (mg/L)** | **NO_2_^-^(mg/L)** | **PO_4_^3-^ (mg/L)** | **Cl^-^(mg/L)** | **SO_4_^2-^(mg/L)** | **K^+^ (mg/L)** | **Ca^2+^ (mg/L)** | **Mz** | **Salinity**  **(ppt)** | **pH** |
| --- | --- | --- | --- | --- | --- | --- | --- | --- | --- | --- | --- | --- |
| QL1 | 11.32 | 2.442 | 0.903 | 1.018 | 0.470 | 1385 | 145.3 | 14.87 | 27.18 | 0.249 | 37.55 | 8.17 |
| QL2 | 6.92 | 1.984 | 0.150 | 0.278 | 0.564 | 1004 | 114.6 | 16.13 | 28.41 | 0.248 | 36.75 | 8.09 |
| QL3 | 16.56 | 1.455 | 0.326 | 0.299 | 0.366 | 1186 | 148.5 | 14.6 | 22 | 0.255 | 36.65 | 8.08 |
| QL4 | 8.73 | 2.256 | 0.698 | 0.288 | 0.617 | 1006 | 135.2 | 12.69 | 25.13 | 0.253 | 36.85 | 8.1 |
| QL5 | 8.02 | 2.083 | 0.205 | 0.251 | 0.564 | 1003 | 103.4 | 13.79 | 22.43 | 0.185 | 39.25 | 8.24 |
| QL6 | 8.40 | 2.089 | 0.095 | 0.107 | 0.105 | 1369 | 129.8 | 17 | 22.76 | 0.331 | 36.25 | 8.09 |
| QL7 | 15.62 | 2.085 | 0.386 | 0.259 | 0.721 | 1062 | 111 | 10.75 | 22.34 | 0.187 | 38.15 | 8.19 |
| QL8 | 8.70 | 1.800 | 0.109 | 0.152 | 0.212 | 874 | 104.9 | 10.83 | 18.6 | 0.332 | 38.35 | 8.16 |
| QM9 | 8.12 | 1.787 | 0.776 | 0.532 | 0.853 | 1178 | 139.4 | 18.6 | 22.89 | 0.181 | 37.45 | 8.11 |
| QM10 | 8.96 | 1.787 | 0.591 | 0.389 | 0.106 | 1071 | 128.8 | 16.12 | 20.75 | 0.253 | 37.3 | 8.11 |
| QM11 | 9.62 | 2.741 | 0.636 | 0.578 | 1.193 | 1437 | 134 | 16.44 | 25.19 | 0.258 | 35.3 | 8.1 |
| QM12 | 8.26 | 2.262 | 0.698 | 0.518 | 1.642 | 1344 | 130.9 | 12.07 | 20.57 | 0.223 | 36.2 | 8.11 |
| QM13 | 6.25 | 2.561 | 0.715 | 0.445 | 3.384 | 1176 | 126.6 | 15.44 | 24.86 | 0.333 | 36.15 | 8.1 |
| QM14 | 7.25 | 2.327 | 0.815 | 0.472 | 0.945 | 1227 | 120.1 | 13.92 | 24.53 | 0.334 | 35.1 | 8.11 |
| QM15 | 2.12 | 2.741 | 0.172 | 0.344 | 0.286 | 1439 | 150.5 | 21.24 | 25.75 | 0.246 | 36.7 | 8.11 |
| QM16 | 7.80 | 1.535 | 0.184 | 0.385 | 0.470 | 1433 | 149 | 32.43 | 31.99 | 0.227 | 36.35 | 8.11 |
| QM17 | 2.75 | 1.987 | 0.205 | 0.450 | 0.523 | 1044 | 121.2 | 20.12 | 23.63 | 0.176 | 37.1 | 8.1 |
| QM18 | 16.25 | 1.840 | 0.326 | 0.410 | 0.052 | 1371 | 136 | 18.14 | 26.54 | 0.185 | 35.95 | 8.09 |
| QM19 | 3.60 | 1.709 | 0.131 | 0.309 | 0.564 | 1277 | 164.8 | 19.9 | 31.45 | 0.271 | 37.05 | 8.11 |
| QM20 | 8.26 | 2.021 | 0.122 | 0.194 | 0.982 | 1287 | 147.5 | 14.29 | 26.45 | 0.257 | 36.85 | 8.1 |
| QM21 | 4.21 | 1.409 | 0.115 | 0.173 | 0.261 | 1040 | 118.9 | 17.35 | 22.13 | 0.266 | 37.2 | 8.17 |
| QH22 | 8.25 | 1.896 | 0.252 | 0.259 | 0.773 | 1381 | 150.6 | 20.36 | 27.55 | 0.168 | 36.05 | 8.08 |
| QH23 | 15.62 | 1.335 | 0.574 | 0.152 | 0.418 | 1119 | 125.3 | 13.76 | 21.33 | 0.246 | 37.4 | 8.18 |
| QH24 | 14.58 | 0.697 | 0.569 | 0.127 | 0.106 | 1380 | 131.4 | 16.38 | 24.36 | 0.243 | 37.25 | 8.16 |
| QH25 | 15.25 | 2.748 | 0.273 | 0.721 | 0.210 | 1235 | 137.2 | 16.69 | 25.9 | 0.179 | 38.25 | 8.16 |
| IL26 | 5.70 | 1.319 | 0.226 | 0.211 | 0.420 | 1102 | 110.3 | 16.8 | 20.16 | 0.195 | 38.6 | 8.18 |
| IL27 | 4.80 | 1.985 | 0.206 | 0.465 | 0.053 | 990 | 98.1 | 12.15 | 20.28 | 0.239 | 37.35 | 8.16 |
| IL28 | 1.02 | 2.010 | 0.206 | 0.450 | 0.470 | 935 | 94.7 | 7.89 | 11.35 | 0.19 | 37.4 | 8.18 |
| IL29 | 2.11 | 1.896 | 0.198 | 0.430 | 0.525 | 1289 | 133 | 15.1 | 24.85 | 0.194 | 36.95 | 8.16 |
| IL30 | 3.30 | 2.256 | 0.184 | 0.384 | 0.106 | 1216 | 143.7 | 16.87 | 21.44 | 0.184 | 37.65 | 8.18 |
| IL31 | 6.62 | 0.489 | 0.115 | 0.173 | 0.107 | 1538 | 144.4 | 25.62 | 27.66 | 0.24 | 37 | 8.14 |
| IM32 | 6.64 | 0.418 | 0.202 | 0.192 | 0.106 | 1598 | 153.4 | 35.02 | 38.67 | 0.17 | 37.35 | 8.15 |
| IH33 | 14.36 | 2.625 | 0.265 | 0.904 | 0.829 | 1364 | 130.8 | 16.07 | 22.79 | 0.197 | 38.25 | 8.17 |
| IH34 | 5.11 | 0.062 | 0.265 | 0.324 | 0.106 | 1247 | 150.2 | 18.75 | 26.26 | 0.279 | 37.35 | 8.14 |
| YL35 | 15.68 | 0.394 | 0.218 | 0.299 | 0.105 | 1215 | 115.2 | 17.05 | 25.77 | 0.335 | 36.95 | 8.18 |
| YM36 | 5.62 | 0.541 | 0.179 | 0.365 | 0.052 | 1197 | 148.8 | 18.01 | 28.17 | 0.195 | 37.7 | 8.18 |
| YM37 | 2.41 | 0.276 | 0.205 | 0.450 | 0.053 | 1543 | 157.2 | 19.89 | 30.02 | 0.178 | 37.35 | 8.19 |
| YM38 | 3.26 | 1.140 | 0.199 | 0.431 | 0.159 | 1380 | 138.4 | 14.63 | 23.2 | 0.196 | 37.75 | 8.18 |
| YM39 | 2.15 | 1.644 | 0.219 | 0.303 | 0.210 | 1379 | 143.5 | 16.43 | 26.26 | 0.185 | 38.8 | 8.18 |

**Table S2.** Biodiversity indexes of bacterial communities.

| Station | Reads | OTUs | Chao | Shannon | Shannoneven | Coverage |
| --- | --- | --- | --- | --- | --- | --- |
| QL1 | 122525 | 106000 | 2922 | 2.69 | 0.36 | 0.97 |
| QL2 | 147259 | 109257 | 4219 | 6.75 | 0.84 | 0.96 |
| QL3 | 159602 | 130509 | 2411 | 5.03 | 0.66 | 0.98 |
| QL4 | 94599 | 69420 | 3291 | 6.35 | 0.81 | 0.97 |
| QL5 | 108685 | 73799 | 3931 | 6.50 | 0.82 | 0.96 |
| QL6 | 57788 | 39678 | 2784 | 6.19 | 0.80 | 0.98 |
| QL7 | 73930 | 59922 | 1972 | 3.92 | 0.53 | 0.98 |
| QL8 | 41595 | 26777 | 2340 | 6.09 | 0.80 | 0.98 |
| QM9 | 53468 | 49192 | 2126 | 1.98 | 0.28 | 0.98 |
| QM10 | 110076 | 79507 | 3811 | 6.10 | 0.77 | 0.96 |
| QM11 | 111781 | 90039 | 2581 | 2.35 | 0.33 | 0.97 |
| QM12 | 141650 | 111077 | 1506 | 1.60 | 0.25 | 0.99 |
| QM13 | 49554 | 43880 | 1612 | 1.84 | 0.28 | 0.98 |
| QM14 | 129397 | 115360 | 1573 | 0.77 | 0.12 | 0.99 |
| QM15 | 47061 | 33827 | 4665 | 6.99 | 0.86 | 0.96 |
| QM16 | 52759 | 35432 | 4527 | 6.58 | 0.81 | 0.96 |
| QM17 | 104348 | 79010 | 4071 | 6.59 | 0.82 | 0.96 |
| QM18 | 113073 | 96669 | 1513 | 2.83 | 0.40 | 0.99 |
| QM19 | 80201 | 52863 | 4360 | 6.96 | 0.86 | 0.96 |
| QM20 | 145350 | 109487 | 4390 | 6.70 | 0.83 | 0.96 |
| QM21 | 95328 | 63947 | 4491 | 6.82 | 0.84 | 0.96 |
| QH22 | 117067 | 88508 | 1942 | 5.48 | 0.74 | 0.98 |
| QH23 | 215660 | 166571 | 3407 | 5.59 | 0.70 | 0.97 |
| QH24 | 90568 | 61278 | 3186 | 5.80 | 0.74 | 0.97 |
| QH25 | 118461 | 104156 | 868 | 2.64 | 0.44 | 0.99 |
| IL26 | 85579 | 54755 | 3403 | 7.16 | 0.89 | 0.98 |
| IL27 | 147377 | 95835 | 2532 | 6.72 | 0.87 | 0.98 |
| IL28 | 174712 | 123586 | 4446 | 7.05 | 0.87 | 0.96 |
| IL29 | 155245 | 113462 | 4334 | 6.98 | 0.86 | 0.96 |
| IL30 | 148727 | 115705 | 4310 | 6.72 | 0.83 | 0.96 |
| IL31 | 97437 | 75411 | 2040 | 6.54 | 0.87 | 0.99 |
| IM32 | 112202 | 96221 | 1956 | 3.01 | 0.41 | 0.98 |
| IH33 | 123697 | 106926 | 624 | 2.71 | 0.44 | 0.99 |
| IH34 | 137526 | 110907 | 2824 | 4.94 | 0.63 | 0.98 |
| YL35 | 107405 | 80458 | 3428 | 5.95 | 0.75 | 0.97 |
| YM36 | 154657 | 114856 | 4132 | 6.66 | 0.83 | 0.96 |
| YM37 | 196594 | 143546 | 4402 | 6.66 | 0.83 | 0.96 |
| YM38 | 76782 | 59586 | 4117 | 6.63 | 0.83 | 0.96 |
| YM39 | 104475 | 79011 | 4410 | 6.91 | 0.85 | 0.96 |

**Table S3.** Genera in different modules from network analysis.

| **Modularity** | **Genus** | **Abundance** | **Modulity** | **Genus** | **Abundance** |
| --- | --- | --- | --- | --- | --- |
| Module1 | g_norank_c_Acidobacteria | 22842 | Module4 | *Fusobacterium* | 3729 |
|  | g_norank_c_Bacteroidetes_BD2.2 | 6993 | Module5 | g_norank_c_Cyanobacteria | 4843 |
|  | g_norank_c_Gammaproteobacteria.1 | 4770 |  | g_unclassified_c_Gammaproteobacteria | 6619 |
|  | g_norank_c_Gemmatimon  .adetes | 19393 |  | g_norank_c_KD4.96 | 4650 |
|  | *Desulfatiglans* | 3464 |  | g_norank_f_DEV007 | 3475 |
|  | g_norank_f_Anaerolineaceae | 11645 |  | g_norank_f_Flammeovirgaceae | 7706 |
|  | g_unclassified_f_Desulfobacteraceae | 4468 |  | g_unclassified_f_Flavobacteriaceae | 3597 |
|  | g_norank_f_Gemmatimonadaceae | 3481 |  | g_norank_f_JTB255_marine_benthic_group | 39047 |
|  | g_norank_f_Rhodospirillaceae | 12133 |  | g_norank_f_OCS116_clade | 4246 |
|  | g_norank_f_Syntrophobacteraceae | 8642 |  | g_norank_f_OM1_clade | 56656 |
|  | *Nitrospira* | 7048 |  | g_norank_f_Sandaracinaceae | 11909 |
|  | g_norank_o_NB1.j | 31196 |  | g_norank_f_Saprospiraceae | 4498 |
|  | g_norank_o_Subgroup_23 | 7395 |  | g_norank_f_Sva0725 | 7727 |
|  | g_norank_o_Sva0485 | 4219 |  | g_norank_f_Sva0996_marine_group | 11253 |
|  | g_norank_p_Aminicenantes | 6328 |  | *Filomicrobium* | 3949 |
|  | g_norank_p_Latescibacteria | 11407 |  | *Granulosicoccus* | 3619 |
|  | *SEEP.SRB1* | 3890 |  | *Ilumatobacter* | 4042 |
|  | *Spirochaeta_2* | 8228 |  | g_norank_o_BD7.8_marine_group | 3831 |
|  | *Sva0081_sediment_group* | 22092 |  | g_norank_o_Gammaproteobacteria_Incertae_Sedis | 4895 |
| Module2 | *Escherichia.Shigella* | 120372 |  | g_norank_o_Rhizobiales | 4229 |
| Module3 | *Bacillus* | 20255 |  | *Urania.1B.19_marine_sediment_group* | 4184 |
|  | g_norank_f_Bacteroidales_S24.7_group | 3907 |  | *Zeaxanthinibacter* | 5463 |
|  | *Lactobacillus* | 7051 | Module6 | *Psychrobacter* | 25726 |
|  | *Lactococcus* | 12707 | Module7 | *Pseudoalteromonas* | 41620 |
|  | *Pseudomonas* | 5844 |  | *Vibrio* | 94188 |
